# Pathological Gait Signatures of Post-stroke Dementia With Toe-Off and Heel-to-Ground Angles Discriminate From Alzheimer’s Disease

**DOI:** 10.3389/fnagi.2021.766884

**Published:** 2021-11-18

**Authors:** Linhui Ni, Wen Lv, Di Sun, Yi Sun, Yu Sun, Xinxin Xu, Mengyue Chang, Xing Han, Shuai Tao, Xingyue Hu, Huaying Cai

**Affiliations:** ^1^Department of Neurology, Sir Run Run Shaw Hospital, School of Medicine, Zhejiang University, Hangzhou, China; ^2^Electroencephalogram Unit, Sir Run Run Shaw Hospital, School of Medicine, Zhejiang University, Hangzhou, China; ^3^Key Laboratory for Biomedical Engineering of Ministry of Education, Department of Biomedical Engineering, Zhejiang University, Hangzhou, China; ^4^Zhejiang Lab, Hangzhou, China; ^5^Dalian Key Laboratory of Smart Medical and Health, Dalian University, Dalian, China

**Keywords:** gait, cognition, post-stroke dementia, Alzheimer’s disease, dual-task gait

## Abstract

Given the limited power of neuropsychological tests, there is a need for a simple, reliable means, such as gait, to identify mild dementia and its subtypes. However, gait characteristics of patients with post-stroke dementia (PSD) and Alzheimer’s disease (AD) are unclear. We sought to describe their gait signatures and to explore gait parameters distinguishing PSD from post-stroke non-dementia (PSND) and patients with AD. We divided 3-month post-stroke patients into PSND and PSD groups based on the Mini-Mental State Examination (MMSE), Montreal Cognitive Assessment (MoCA), and the activity of daily living (ADL). Thirty-one patients with AD and thirty-two healthy controls (HCs) were also recruited. Ten gait parameters in one single and two dual-task gait tests (counting-backward or naming-animals while walking) were compared among the groups, with adjustment for baseline demographic covariates and the MMSE score. The area under the receiver operating characteristic curve (AUC) was used to identify parameters discriminating PSD from individuals with PSND and AD. Patients with PSD and patients with AD showed impaired stride length, velocity, stride time, and cadence while patients with PSD had altered stance and swing phase proportions (all *p* ≤ 0.01, *post hoc*). Patients with AD had smaller toe-off (ToA) and heel-to-ground angles (HtA) (*p* ≤ 0.01) than HCs in dual-task gait tests. Individuals with PSD had a shorter stride length, slower velocity, and altered stance and swing phase percentages in all tests (*p* ≤ 0.01), but a higher coefficient of variation of stride length (CoV_SL_) and time (CoV_ST_) only in the naming animals-task gait test (*p* ≤ 0.001) than individuals with PSND. ToA and HtA in the naming animals-task gait test were smaller in individuals with AD than those with PSD (*p* ≤ 0.01). Statistical significance persisted after adjusting for demographic covariates, but not for MMSE. The pace and the percentage of stance or swing phase in all tests, CoV_ST_ in the dual-task paradigm, and CoV_SL_ only in the naming animals-task gait test (moderate accuracy, AUC > 0.700, *p* ≤ 0.01) could distinguish PSD from PSND. Furthermore, the ToA and HtA in the naming animals-task gait paradigm discriminated AD from PSD (moderate accuracy, AUC > 0.700, *p* ≤ 0.01). Thus, specific gait characteristics could allow early identification of PSD and may allow non-invasive discrimination between PSD and AD, or even other subtypes of dementia.

## Introduction

Approximately, 50 million people suffer from dementia (WHO, 2018). It is one of the major causes of disability and mortality among aging adults (WHO, 2018). The two most common types of dementia are Alzheimer’s disease (AD) and vascular dementia (VaD) caused by stroke (WHO, 2018; Emrani et al., 2020). Dementia is characterized by amnesia, impaired executive function, visuospatial capacity, and attention. Currently, diagnosis depends on the temporal relationship of the disease symptoms with imaging examination findings and neuropsychological tests (Emrani et al., 2020; Ismail et al., 2020). However, early diagnosis of post-stroke dementia (PSD) and mild AD is difficult because of the limited sensitivity of the cognitive function scales, particularly under repetitive interview conditions. Additionally, educational attainment, cultural background, and even hearing or speaking abilities may reduce the specificity of the neuropsychological tests. Overlapping symptoms and imaging manifestations, multifactorial causes, and homogeneity of the histopathology limit the accuracy of distinguishing among dementia subtypes. Pathological biopsy of brain tissue is the gold standard for dementia classification, but this is not generally applicable due to its invasive nature (Schott et al., 2010). There is no specific biomarker that can robustly identify vulnerable patients with PSD from patients with post-ischemic stroke or non-PSD dementia subtypes. Thus, there is a need to identify safe, reliable, and effective clinical markers to enhance diagnostic accuracy.

The human gait is remarkably complex. Gait in older people is divided into five primary modal domains: pace, rhythm, variability, asymmetry, and postural control (Lord et al., 2013). An integrated gait reflects the health of individuals, particularly in compensating for changes in postural balance and preventing falls. This is controlled by well-balanced neural circuits and specific brain structures involving the frontal and limbic regions, basal ganglia, cerebellum, and optical, vestibular, sensory, and motor systems (Takakusaki, 2013; Tian et al., 2017; Allali et al., 2019). Memory, attention, executive function, and visual-spatial capacity share some overlapping brain regions related to gait (Morris et al., 2016). Therefore, gait is no longer regarded as a purely autonomic movement. A healthy integrated gait requires attention, executive function, and visual and auditory capacities. Spatiotemporal gait characteristics in the single-gait test of cerebrovascular disease and neurodegenerative diseases have been described, particularly for cases with mild cognitive impairment (MCI), AD, and Parkinson’s disease (PD) (Mc Ardle et al., 2019; Sanders et al., 2020). In recent years, studies have increasingly implemented the dual-task gait paradigm, which requires subjects to walk while accomplishing an additional cognitive task, to reflect the cognitive challenges at the cognitive-motor interface, increasing the sensitivity for discovering occult cognitive deterioration (Bayot et al., 2018).

Gait stride length and velocity, belonging to the pace domain of gait, have been assessed most commonly in this field, because of the ease of acquisition. The decreased pace and increased instability have been detected in the general older population (Cohen et al., 2016; Noce Kirkwood et al., 2018; Rasmussen et al., 2019). It is controversial whether individuals with MCI show gait dysfunction as compared to matched healthy aging adults. In addition, MCI sufferers who walk slower and who demonstrate higher dual-task costs have been shown to be at risk of progression to dementia (Montero-Odasso et al., 2017). Recently, numerous cohort studies have shown weaker gaits in patients with AD, manifested as decreased pace, greater variability, and worse rhythm in the normal gait test, and pathological gait parameters would be more sensitive measured during dual-task gait measurements (Mc Ardle et al., 2017, 2019). Moreover, evidence suggests that the asymmetry increases with cognitive decline (Ghoraani et al., 2021). There were rarely differences found in postural control during walking between patients with AD and age-matched healthy adults or those with other cognitive impairments (Gillain et al., 2009; Maquet et al., 2010).

In terms of discrete gait characteristic comparisons among dementia subtypes, reports have outlined distinctive patterns of gait damage under a few dual-task gait measurements. Differences in gait damage in individuals with AD and those with non-AD dementia had frequently been reported, mostly in the late stage of AD. Patients with AD showed less impairment in pace, rhythm, and variability than those with non-AD dementia, such as fronto-temporal dementia and Lewy body dementia (Beauchet et al., 2016). People with VaD showed a poorer pace than patients with AD (Allan et al., 2005). However, there are few studies available on domains of gait other than pace for distinguishing between VaD and AD. There have been rare descriptions of differences in gait between individuals with PSD, a subtype of VaD, and those with AD, and there is no specific gait-based predictor that can identify early dementia in post-stroke patients. Early recognition of PSD in patients with ischemic stroke, at 3 months from stroke initiation, is crucial for the follow-up treatment strategy and predicting prognosis, because of the probable remission and even reversion of PSD (van der Flier et al., 2018). On the other hand, specific subtypes of dementia require targeted therapy to control disease progression. Thus, the early and definite typology of dementia is of great clinical significance. In recent years, studies of machine learning, based on using data from wearable devices to detect spatiotemporal gait parameters, have increased and have been confirmed as an effective method for measuring the relationship between gait parameters and neurological functions (Cheng et al., 2020). The inconsistent cognitive condition of post-stroke patients would place varying degrees of cognitive load on gait performance. We hypothesized that this might be differences in spatiotemporal gait parameters between states of dementia (PSD) and non-dementia post-stroke (PSND). On the other hand, discrete pathologies and cognition formation may result in unique gait patterns between individuals with PSD and those with AD.

The goal of this study was to elucidate a motion marker that could identify PSD in ischemic stroke patients, and to outline the typical gait features in individuals with PSD and those with AD, providing a low-cost, feasible, and effective means for earlier detection of PSD or non-PSD subtypes of dementia in order to implement interventions for cognitive impairment as early as possible. Thus, in the present study, we compared spatiotemporal gait patterns and neuropsychiatric parameters among healthy older individuals (HCs) and age-matched individuals with PSD, PSND, or AD. To this end, we introduced parameters, i.e., the toe-off angle (ToA) and heel-to-ground angle (HtA), respectively, measured at the moment of initiation or end of the swing phase, which has not been reported in previous studies of PSD and AD, to the best of our knowledge. Respective comparisons of PSD with PSND or AD were performed to clarify gait differences that allowed the distinction of these conditions.

## Participants and Methods

### Study Design and Participants

This clinical study is a monocentric cross-sectional study. Sixty-six outpatients at 3-month post ischemic stroke and 31 patients with mild to moderate AD were recruited from the Clinic of the neurology department, Sir Run Run Shaw Hospital of Zhejiang University in China, and 32 matched healthy control subjects (HCs) were recruited from the Clinic or physical examination center. The stroke patients were first-episode with definitive acute ischemic based on MRI, and in normal cognition before the stroke. They were then categorized into the group of PSD or PSND depending on the cognitive and activity of daily living assessments, and related clinical presentations. The stroke related scales of recruited samples were defined as follows: the National Institution Health of Stroke Scale (NIHSS) ≤4, the Modified Rankin Scale (mRS) ≤2, and the muscle strength ≥ 4+ grade.

All participants had to be aged over 55 years old and can walk at least 10 m without any assistant. We will exclude adults who: can’t speak fluently; with Parkinsonism symptoms or other neurological diseases influencing cognition or gait (such as PD dementia with Lewy bodies, frontal–temporal dementia, and dystonia); with osteoarticular diseases which might influence on walking; and/or with severe mental illness, such as major depression (total score > 7 on the 17-item Hamilton Depression Rating Scale), anxiety (total score > 7 on Hamilton Anxiety Rating Scale), bipolar disorder, and schizophrenia, or any psychotropic drugs taken.

### Clinical and Cognitive Assessment

At all follow-up visits, participants had an interview with the same neurology doctor to complete the demography baseline information collection including age, gender, education level, height, weight, comorbidities, and habits of smoking and drinking. The interviewers combined the results of medical history and imaging reports, with neurological examination to confirm whether the subject enrolled. The depression condition was evaluated using the 17-item Hamilton Depression Rating Scale (HAMD), and the anxiety condition was measured by Hamilton Anxiety Rating Scale (HAMA). The Activity of Daily Living Scale (ADL) was used to assess the self-care ability of patients in daily life. The baseline and 3 months post stroke of NIHSS and mRS of ischemic stroke patients were assessed, with the muscle strength and lesion side reported by MRI were also collected.

Post-stroke dementia was diagnosed by the two same neurology doctors according to the 2019 Chinese Vascular Cognitive Impairment Guideline, which defined PSD as a status with cognitive impairment and impaired activity of daily living lasting for 3 months after stroke onset (Cognitive Impairment Committee Nb, Chinese Medical Doctor Association, 2019). We, therefore, determined the enrolled stroke patients in 3 months post stroke, which is also consistent with the international consensus (within 6 months) (Skrobot et al., 2018). All patients with AD met the 2011 revised criteria for AD diagnosis of National Institute on Aging-Alzheimer’s Association diagnostic guidelines (Khachaturian, 2011). The clinical and MRI data of all patients are available.

The cognitive assessments included the Mini-Mental State Examination (MMSE), Montreal Cognitive Assessment (MoCA), and Mini-Cog. On the basis of Chinese national conditions and education levels of the general elderly adults, we defined the cognitive impairment assessed by MMSE to illiteracy ≤19, primary school ≤22, middle school and above ≤26 (Li et al., 2016), MoCA to illiteracy ≤13, primary school ≤19, middle school and above ≤24 (Lu et al., 2011), and Mini-Cog ≤3 (McCarten et al., 2011).

### Gait Testing Procedure

All gait assessments were performed in a spacious hallway outside the clinic room using wearable motion sensors (JiBuEn^R^ gait analysis system, version 2.3). Patients walked at least 10 m at their comfortable pace with or without a cognitive task. Five steps of each start and end of the pathway were deleted to ensure the acceleration and deceleration phases were not recorded. All participants were asked to accomplish three gait trials. First, participants were asked to walk at their normal, everyday walking speed. The next two dual-task gait tests comprised walking while counting backward, or naming animals, which have been validated in previous clinical trials that robustly increase cognitive demand of adults (Montero-Odasso et al., 2017). Only participants who completed all three trials were included in the analysis. Spatiotemporal gait parameters representing pace (stride length and velocity), rhythm (stride time, cadence, percentages of stance and swing phase), variability [coefficient of variation (CoV) of stride length (CoV_SL_) and stride time (CoV_ST_)], and postural control involving ToA and HtA were collected. The CoV was calculated as follows:


CoV(%)=SDofparameter/Meanofparameter×100%

**FIGURE 1:**
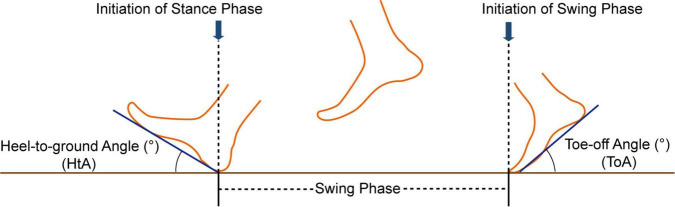
The toe-off angle (ToA) and heel-to-ground angle (HtA) in the gait cycle.

Toe-off angle was defined as the angle of toe-off the ground measured at the moment of initiation of the swing phase. HtA was defined as the angle of initial heel stride to the ground measured at the moment of initiation of the stance phase (Figure 1).

### Covariates

Analyses were adjusted for covariates that included demographics (age, gender, education levels, and height), numbers of comorbidity, and baseline cognition (MMSE). In a comparison of PSD and PSND, muscle strength was added to the covariates.

### Statistical Analysis

The baseline demography and clinical information, cognitive assessments, and gait parameters of four groups were displayed through descriptive analysis. We evaluated the data distribution of all quantitative variables in each group using the one-sample Kolmogorov–Smirnov test and histograms. Variables that had normal distribution will be described using means and SD, while variables with non-normal distribution were presented using median and inter-quartile range (IQR). Categorical variables were described by frequencies and percentages.

Physical information, cognitive scores, and gait parameters comparisons among four groups were analyzed using a one-way ANOVA test when quantitative variables were in normal distribution and homoscedasticity, otherwise, they were analyzed using the Kruskal–Wallis *H* test. The chi-square test was used to determine the categorical data. The LSD analysis was used for *post hoc* analysis, of which, significance was defined as *p* ≤ 0.01. Student *t*-test was assessed to compare the gait parameters of PSD with PSND or AD group, respectively, yet the pairwise analysis was using Mann–Whitney *U* test if data in non-normal distribution or non-homoscedasticity. General linear regression was used to control for primary demography covariates of age, gender, education levels, height, and numbers of comorbidity with or without MMSE when data in normal distribution and homoscedasticity. Otherwise, generalized linear regression was used. The parameter under *p* < 0.05 was determined as the potential predictor of PSD from AD. To more precisely identify the PSD from patients with stroke, the significance was shrinking to a more conservative threshold of *P* ≤ 0.01. Moreover, stepwise logistic regression was used to verify the superiority of the above gait predictor for identifying PSD. The receiver operating characteristic curve analysis (ROC) and area under the curve (AUC) determined the overall accuracy of possible distinguisher for PSD from individuals with ischemic stroke or AD.

## Results

### Baseline Information

A total of 127 subjects (32 HCs, 32 PSND, 32 PSD, and 31 AD participants) were included in this longitudinal study. The baseline characteristics of participants, including medical and cognitive conditions, are summarized in Table 1. The numbers of comorbidities (χ^2^ = 27.22, *p* ≤ 0.001) and ratios of hypertension (χ^2^ = 26.8, *p* ≤ 0.001) were significant among the four groups. The age, female proportion, education level, height, and proportion of those with diabetes, smoking and drinking habits, and depression and anxiety conditions were not significantly different (*p* > 0.05). The activities of daily living of PSD and AD were significantly different from HCs and PSND, respectively (*p* ≤ 0.001, *post hoc*, Table 1). The NIHSS, mRS, muscle strength, and infarcted lesion side were not significantly different between PSD and PSND patients (*p* > 0.05, Supplementary Table 1).

**TABLE 1:** Baseline characteristics of participants stratified by included samples.

	HCs	PSND	PSD	AD	F/χ2	*P*
**Characteristics**	*n* = 32	*n* = 32	*n* = 32	*n* = 31		
Age, mean (SD)	67.7 (5.13)	67.8 (6.53)	68.4 (8.44)	71.8 (8.23)	2.08	0.106
Female, no. (%)	19 (59.4)	11 (34.4)	12 (37.5)	18 (58.1)	6.69	0.082
Education level, no. (%)					10.46	0.106
Illiterate	2 (6.3)	8 (25.0)	10 (31.3)	5 (16.1)		
Primary school	7 (21.9)	8 (25.0)	9 (28.1)	5 (16.1)		
Middle school and above	23 (71.9)	16 (50.0)	13 (40.6)	21 (67.7)		
Height, mean (SD)	161.6 (5.65)	166.0 (7.27)	164.3 (6.71)	161.5 (5.88)	2.60	0.056
No. of comorbidities, median (IQR)	1.0 (2.0)	2.0 (1.0)	2.0 (2.0)	1.5 (2.0)	27.22	**<0.001**
Comorbidities, no. (%)						
Hypertension	11 (34.4)	25 (78.1)	29 (90.6)	16 (51.6)	26.80	**<0.001**
Diabetes	6 (18.8)	10 (31.3)	9 (28.1)	6 (19.4)	2.04	0.565
Smoking, no. (%)	10 (31.3)	15 (46.9)	14 (43.8)	8 (25.8)	4.08	0.253
Drinking, no. (%)	4 (12.5)	8 (25.0)	10 (31.3)	6 (19.4)	3.57	0.312
HAMD, median (IQR)	3.0 (3.0)	2.0 (4.0)	3.0 (5.0)	4.0 (3.0)	1.66	0.645
HAMA, median (IQR)	2.0 (4.0)	3.0 (4.0)	3.0 (5.0)	3.0 (5.0)	5.78	0.123
ADL, median (IQR)	14.0 (0)^S,A^	14.19 (0)	20.5 (7.0)^N^	20.3 (12.0)	103.03	**<0.001**

**Cognition assessment**					***P***	**Adj. *P***

MMSE, median (IQR)	27.0 (2.0)^S,A^	27.0 (4.0)	22.5 (10.0)^N^	17.0 (11.0)	**<0.001**	**<0.001**
MoCA, median (IQR)	25.0 (3.0)^S,A^	25.0 (4.0)	16.5 (7.0)^N^	13.0 (10.0)	**<0.001**	**<0.001**
Mini-Cog, median (IQR)	5.0 (1.0)^S,A^	5.0 (1.0)	2.0 (2.0)^N,A^	1.0 (2.0)	**<0.001**	**<0.001**

*Data of continuous variables described as means (SD) were assessed using One-way ANOVA analysis, whereas data displayed as median (IQR) were used Kruskal–Wallis H tests. Data of categorical variables were described by frequencies and percentages using the chi-square test. Bold values highlight the significant difference. Adj. P, P-value when adjusting for education level; S, different to PSD; A, different to AD; N, different to PSND; HCs, healthy controls; PSND, post-stroke non-dementia; PSD, post-stroke dementia; AD, Alzheimer’s disease; BMI, body mass index; HAMD, 17-item Hamilton Depression Rating Scale; HAMA, Hamilton Anxiety Rating Scale; ADL, Activity of Daily Living Scale; MMSE, Mini-mental State Examination; MoCA, Montreal Cognitive Assessment.*

The MMSE, MoCA, and Mini-Cog scores were significantly different among the four groups after controlling for education level (MMSE: Wald χ^2^ = 180.686, *p* ≤ 0.001; MoCA: Wald χ^2^ = 365.823, *p* ≤ 0.001; Mini-Cog: Wald χ^2^ = 220.423, *p* ≤ 0.001, Table 1). PSD and AD groups showed lower MMSE, MoCA, and Mini-Cog scores than the HCs and PSND groups (*p* ≤ 0.01, *post hoc*, Table 1), and the AD group had lower Mini-Cog scores than the PSD group (*p* ≤ 0.01, *post hoc*, Table 1). The gait characteristics of the four gait domains among the four groups are presented in Table 2.

**TABLE 2:** Comparison of gait characteristics among controls, non-dementia post-stroke, and dementia subtypes.

	HCs	PSND	PSD	AD	*P*	Adj. *P*
**Pace**						
**Stride length (m)**						
Single-task	1.18 (0.15)^S,A^	1.20 (0.16)	1.05 (0.31)^N^	1.06 (0.24)	**0.001**	**<0.001**
Counting	1.16 (0.13)^S,A^	1.24 (0.10)	1.05 (0.26)^N^	1.08 (0.32)	**<0.001**	**<0.001**
Naming animals	1.12 (0.19)^S,A^	1.18 (0.19)	1.00 (0.29)^N^	1.00 (0.35)	**<0.001**	**<0.001**
**Velocity (m/s)**						
Single-task	1.10 (0.18)^S,A^	1.05 (0.20)	0.96 (0.28)^N^	0.91 (0.27)	**<0.001**	**<0.001**
Counting	1.08 (0.18)^S,A^	1.07 (0.18)	0.92 (0.36)^N^	0.88 (0.33)	**<0.001**	**<0.001**
Naming animals	0.98 (0.12)^S,A^	0.92 (0.24)	0.72 (0.23)^N^	0.70 (0.53)	**<0.001**	**<0.001**
**Variability**						
**CoV_SL_ (%)**						
Single-task	3.99 ± 1.35^S,A^	4.37 ± 1.18	5.26 ± 1.74	5.96 ± 2.00	**<0.001**	**<0.001**
Counting	4.41 (1.81)^A^	5.35 (1.59)	5.76 (2.61)	5.20 (3.91)	0.066	**0.031**
Naming animals	5.32 (3.96)^S,A^	5.07 (1.56)	7.16 (4.74)^N^	8.64 (4.69)	**<0.001**	**<0.001**
**CoV_ST_ (%)**						
Single-task	2.34 ± 1.06	2.35 ± 0.84	3.10 ± 1.67	3.13 ± 1.19	**0.010**	0.103
Counting	2.22 (2.89)	1.95 (1.15)	3.24 (1.49)^N^	2.75 (1.80)	**0.005**	0.246
Naming animals	5.00 (5.10)	3.84 (2.19)	6.13 (4.00)^N^	6.67 (6.73)	**<0.001**	**<0.001**
**Rhythm**						
**Stride time (s)**						
Single-task	1.06 (0.07)^S,A^	1.11 (0.09)	1.16 (0.13)	1.15 (0.21)	**<0.001**	**<0.001**
Counting	1.08 (0.13)^S,A^	1.23 (0.05)	1.19 (0.18)	1.17 (0.25)	**<0.001**	**<0.001**
Naming animals	1.12 (0.15)^S,A^	1.22 (0.20)	1.39 (0.35)	1.34 (0.24)	**<0.001**	**<0.001**
**Cadence (steps/min)**						
Single-task	113.74 (7.56)^S,A^	108.11 (8.88)	103.00 (12.75)	104.35 (18.09)	**<0.001**	**0.001**
Counting	110.09 (13.25)^S,A^	106.67 (4.90)	100.84 (16.02)	102.56 (18.56)	**0.001**	**0.014**
Naming animals	101.93 (9.90)^S,A^	96.00 (16.26)	85.41 (20.83)	86.96 (13.55)	**<0.001**	**<0.001**
**Stance phase (%)**						
Single-task	62.07 ± 1.70^S^	62.15 ± 1.77	63.95 ± 2.62^N^	63.09 ± 2.43	**0.002**	**0.014**
Counting	62.64 ± 2.03^S^	62.49 ± 1.33	64.50 ± 2.79^N^	63.56 ± 1.98	**0.001**	**0.009**
Naming animals	64.90 ± 2.49^S^	64.89 ± 2.34	67.56 ± 4.06^N^	66.84 ± 3.10	**0.001**	**0.009**
**Swing phase (%)**						
Single-task	37.93 ± 1.70^S^	37.86 ± 1.77	36.04 ± 2.62^N^	36.91 ± 2.44	**0.002**	**0.013**
Counting	37.36 ± 2.03^S^	37.51 ± 1.33	35.52 ± 2.80^N^	36.45 ± 1.98	**0.001**	**0.010**
Naming animals	35.12 ± 2.51^S^	35.12 ± 2.35	32.45 ± 4.06^N^	33.17 ± 3.11	**0.001**	**0.009**
**Postural control**						
**Toe-off angle (°)**						
Single-task	46.05 (5.76)^A^	43.69 (20.76)	39.10 (25.74)	36.41 (30.27)	**0.002**	**0.009**
Counting	46.05 (7.97)^A^	43.64 (27.64)	39.35 (26.20)	35.65 (31.38)	**0.007**	0.106
Naming animals	43.98 (7.27)^S,A^	39.69 (22.91)	35.06 (24.65)	16.30 (25.38)	**<0.001**	**<0.001**
**Heel-to-ground angle (°)**						
Single-task	34.17 (4.81)	33.90 (23.19)	26.43 (18.36)	29.85 (23.88)	**0.008**	0.051
Counting	33.03 (6.06)	31.08 (25.90)	28.35 (20.41)	30.55 (23.50)	0.176	0.666
Naming animals	30.43 (6.47)^A^	28.40 (22.01)	25.18 (16.92)	14.17 (8.60)	**<0.001**	**<0.001**

*Data of continuous variables described as means ± SD were assessed using One-way ANOVA analysis if normal distributed and homogeneity, whereas displayed as median (IQR) and were used Kruskal–Wallis H test. The post hoc is to compare each group with every other group, respectively. The significant difference assessed by post hoc defined p ≤ 0.01 and was marked in the top right corner of the parameter result. Bold values highlight the significant difference among the four groups. The adjusted modal is controlling for age, gender, education levels, height, and numbers of comorbidity. S, different to PSD; A, different to AD; N, different to PSND; HCs, healthy controls; PSND, post-stroke non-dementia; PSD, post-stroke dementia; AD, Alzheimer’s disease; CoV, coefficient of variation.*

### Gait Impairment in the Dementia Subtypes (Alzheimer’s Disease and Post-stroke Dementia) Compared With Healthy Controls

Compared to HCs, patients with PSD and AD had a shorter stride length, slower gait velocity, decreased cadence, and longer stride time in single or dual-task gait tests (*p* ≤ 0.01, *post hoc*, Table 2). In the PSD group, the percentage of time spent in the stance phase was longer and that spent in the swing phase was shorter in all gait tests, while disturbed CoV_ST_ and HtA were observed only in the naming animals-task gait test (*p* ≤ 0.01, *post hoc*, Table 2). We observed greater CoV_SL_ and smaller ToA and HtA in the AD than in the HCs group in all gait paradigms.

### Gait Impairment in Post-stroke Dementia Compared to Post-stroke Non-dementia Individuals

**FIGURE 2:**
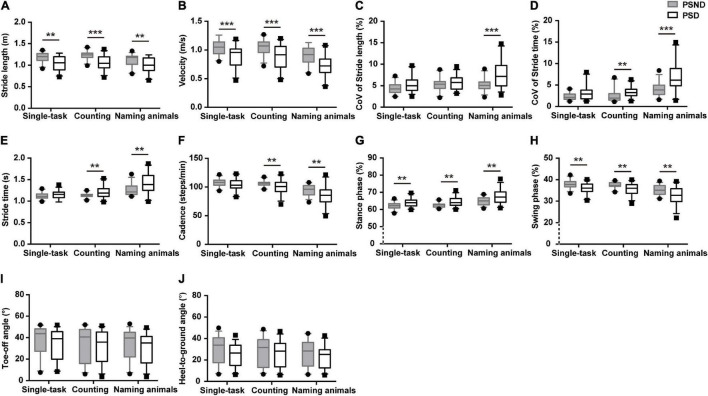
The comparisons of spatiotemporal gait characteristics between individuals with post-stroke non-dementia (PSND) and individuals with post-stroke dementia (PSD) in three gait paradigms. **(A,B)** The pace domain of stride length and velocity. **(C,D)** The variability domain of coefficient of variation (CoV) of stride length (CoV_SL_) and stride time (CoV_ST_). **(E–H)** The rhythm domain of stride time, cadence, and percentages of the stance and swing phases. **(I,J)** The ToA and HtA of PSD and PSND individuals. **p ≤ 0.01, ***p ≤ 0.001.

Patients with ischemic stroke showed no difference in NIHSS score, mRS score, muscle strength, or lesion side when they returned to the clinic at the third month post-stroke (*p* > 0.05, Supplementary Table 1). The spatiotemporal gait parameters of individuals in the PSND group were not different from those of HCs (*p* > 0.05, *post hoc*, Table 2). However, a worsening pace and disturbed gait phase of individuals with PSD were noted in the single and dual-task gait tests (*p* ≤ 0.01, Table 3 and Figure 2). Individuals with PSD showed increased CoV_ST_, stride time, and decreased cadence during dual-tasks, while increased CoV_SL_ was only observed in this group during the naming animals-task gait test (*p* ≤ 0.01, Table 3 and Figure 2). The differences remained robust after controlling for primary baseline covariates (age, gender, education levels, height, and numbers of comorbidity, modal 1 of Table 3), except for the CoV_ST_ and stride time during the counting backward-task, and cadence in the two dual-task gait paradigms (*p* ≤ 0.01, Table 3). The differences were no longer significant after additional adjustment for MMSE scores and other primary baseline (age, gender, education levels, height, and numbers of comorbidity, modal 2 of Table 3) (*p* > 0.01, Table 3).

**TABLE 3:** Comparison of gait characteristics of individuals with post-stroke dementia (PSD) and individuals with post-stroke non-dementia (PSND).

	Unadjusted model		Adjusted model 1		Adjusted model 2	
	t/U	*P*	F/χ^2^	*P*	F/χ^2^	*P*
**Pace**						
**Stride length (m)**						
Single-task	256.5	**0.001**	14.060	**<0.001**	1.675	0.196
Counting	4.967	**<0.001**	23.278	**<0.001**	6.231	0.013
Naming animals	263.0	**0.004**	7.548	**0.006**	4.595	0.032
**Velocity (m/s)**						
Single-task	3.769	**<0.001**	10.832	**0.002**	1.939	0.170
Cunting	4.051	**<0.001**	11.016	**0.002**	4.027	0.045
Naming animals	3.875	**<0.001**	12.968	**0.001**	6.032	0.018
**Variability**						
**CoV_SL_ (%)**						
Single-task	−2.265	0.027	4.943	0.031	3.234	0.079
Forward counting	−0.914	0.364	0.267	0.608	0.139	0.711
Naming animals	−4.213	**<0.001**	13.161	**<0.001**	1.588	0.208
**CoV_ST_ (%)**						
Single-task	−2.204	0.033	3.330	0.074	2.668	0.109
Counting	623.0	**0.001**	6.235	0.013	3.867	0.049
Naming animals	−4.369	**<0.001**	16.292	**<0.001**	11.409	**0.001**
**Rhythm**						
**Stride time (s)**						
Single-task	541.0	0.266	1.158	0.282	1.228	0.268
Counting	−2.974	**0.005**	5.575	0.018	2.112	0.146
Naming animals	639.0	**0.005**	7.824	**0.005**	3.713	0.054
**Cadence (steps/min)**						
Single-task	1.699	0.094	2.086	0.155	1.163	0.286
Counting	2.957	**0.005**	5.195	0.023	1.960	0.161
Naming animals	2.752	**0.009**	5.831	0.016	0.957	0.328
**Stance phase (%)**						
Single-task	−3.163	**0.003**	7.285	**0.009**	3.569	0.065
Counting	−3.649	**0.001**	10.191	**0.001**	4.370	0.037
Naming animals	−3.216	**0.002**	7.455	**0.006**	0.685	0.408
**Swing phase (%)**						
Single-task	3.198	**0.002**	7.402	**0.009**	3.626	0.063
Counting	3.604	**0.001**	9.912	**0.002**	4.211	0.040
Naming animals	3.212	**0.002**	7.431	**0.006**	0.668	0.414
**Postural control**						
**Toe-off angle (°)**						
Single-task	409.0	0.167	0.486	0.486	1.079	0.299
Counting	408.5	0.165	0.077	0.781	0.253	0.615
Naming animals	387.5	0.136	1.315	0.251	1.600	0.206
**Heel-to-ground angle (°)**						
Single-task	362.5	0.045	2.302	0.129	4.346	0.037
Counting	434.0	0.295	0.157	0.694	0.179	0.674
Naming animals	1.256	0.214	1.135	0.021	1.159	0.287

*Normally distributed data used Student’s t-test, and control for primary covariates by general linear models, otherwise used Mann–Whitney U test, and control for primary covariates by generalized linear models. The significant difference is confined by p ≤ 0.01. Bold values highlight the significant differences between the two groups. Adjusted model 1: Controlling for age, gender, education levels, height, muscle strength, and numbers of comorbidity. Adjusted model 2: Controlling for age, gender, education levels, height, muscle strength, numbers of comorbidity, and MMSE.*

**FIGURE 3:**
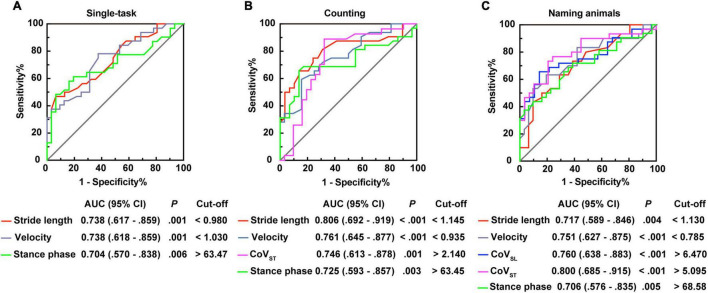
Receiver operating characteristic curve analysis (ROC) plots for gait characteristics identifying the individuals with post-stroke dementia (PSD) from individuals with post-stroke non-dementia (PSND). **(A)** Stride length, velocity, and percentages of the stance phase for identifying patients with PSD in the single-task gait test. **(B)** Stride length, velocity, CoV_ST_, and percentages of the stance phase for distinguishing patients with PSD in the counting backward-task gait test. **(C)** Stride length, velocity, CoV_SL_, CoV_ST_, and percentages of the stance phase for distinguishing patients with PSD in the naming animals-task gait test. AUC, area under the curve; CI, confidence interval; cut-off, cut-off point; CoV, coefficient of variation.

Moreover, stepwise logistic regression validated the importance of the above parameters to distinguish patients with PSD from that of PSND (*p* < 0.05, Table 4), and the AUCs showed that stride length, velocity, and the percentage of time spent in the stance or swing phase in the counting-task gait test showed moderate accuracy for distinguishing PSD from PSND individuals (AUCs ≥ 0.725, Figure 3). The CoV_ST_ in the naming animals-gait test might be optimal for recognizing subjects with PSD from individuals with PSND [AUC = 0.800 (0.685–0.915), *p* ≤ 0.001, Figure 3].

**TABLE 4:** Logistic regression of primary gait parameters to identify individuals with PSD from individuals with PSCN.

	Unadjusted model			Adjusted model 1			Adjusted model 2		
	OR	95% CI	*P*	OR	95% CI	*P*	OR	95% CI	*P*
**Single-task**									
Stride lengths	0.001	0.0–0.08	**0.002**	0.0	0.0–0.06	**0.003**	0.031	0.0–17.81	0.283
Velocity	0.003	0.0–0.12	**0.002**	0.001	0.0–0.07	**0.002**	0.003	0.0–1.77	0.074
Stance phase	1.46	1.11–1.92	**0.006**	1.95	1.28–2.96	**0.002**	2.33	1.11–4.90	0.026
Swing phase	0.683	0.52–0.90	**0.006**	0.512	0.34–0.80	**0.002**	0.430	0.21–0.90	0.026
**Counting**									
Stride lengths	0.0	0.0–0.02	**0.001**	0.0	0.0–0.003	**0.001**	0.0	0.0–1.76	0.064
Velocity	0.003	0.0–0.11	**0.001**	0.0	0.0–0.03	**0.001**	0.0	0.0–0.70	0.040
CoV_ST_	1.62	1.06–2.48	0.027	1.87	1.10–3.19	0.022	3.40	1.12–10.30	0.031
Stride time	1.01	1.00–1.01	0.017	1.01	1.00–1.02	0.021	1.02	0.999–1.04	0.069
Cadence	0.92	0.86–0.99	0.018	0.900	0.83–0.97	**0.009**	0.810	0.66–1.00	0.051
Stance phase	1.57	1.15–2.13	**0.004**	1.91	1.28–2.85	**0.002**	2.44	0.88–6.71	0.085
Swing phase	0.643	0.47–0.87	**0.005**	0.530	0.36–0.79	**0.002**	0.429	0.16–1.14	0.429
**Naming animals**									
Stride lengths	0.006	0.0–0.30	0.011	0.004	0.0–0.36	0.016	0.003	0.0–10.32	0.164
Velocity	0.003	0.0–0.13	**0.002**	0.002	0.0–0.10	**0.002**	0.0	0.0–0.44	0.032
CoV_SL_	1.62	1.18–2.21	**0.003**	1.67	1.20–2.34	**0.003**	1.60	0.97–2.64	0.068
CoV_ST_	1.73	1.24–2.41	**0.001**	1.94	1.27–2.95	**0.002**	4.62	1.34–15.98	0.016
Stride time	1.00	1.001–1.008	**0.007**	1.01	1.001–1.008	**0.008**	1.01	1.001–1.02	0.031
Cadence	0.948	0.91–0.99	0.016	0.947	0.91–0.99	0.019	0.89	0.80–0.99	0.030
Stance phase	1.28	1.07–1.54	**0.008**	1.30	1.07–1.58	**0.010**	1.53	0.99–2.36	0.057
Swing phase	0.781	0.65–0.93	**0.008**	0.771	0.63–0.94	**0.010**	0.662	0.43–1.02	0.058

*0.0 means the data is greater than but infinitely close to 0. Bold values highlight the significant differences between the two groups. Adjusted model 1: Controlling for age, gender, education levels, height, muscle strength, and numbers of comorbidity. Adjusted model 2: Controlling for age, gender, education levels, height, muscle strength, numbers of comorbidity, and MMSE.*

### Differences in Gait Parameters Between Post-stroke Dementia and Alzheimer’s Disease Individuals

**FIGURE 4:**
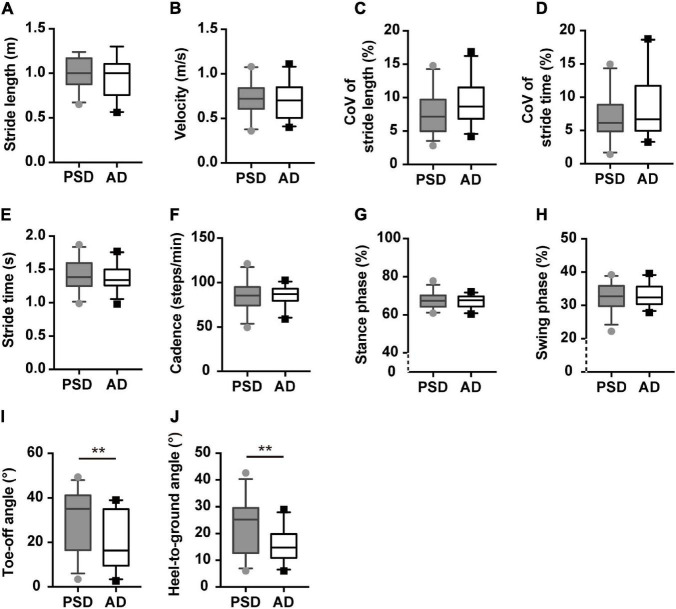
Gait characteristics comparisons of patients with PSD and patients with AD showed in the naming animals-task gait test. **(A,B)** The pace domain of stride length and walking speed. **(C,D)** The variability domain of CoV_SL_ and CoV_ST_. **(E–H)** The rhythm domain of stride time, cadences, percentages of stance, and swing phases. **(I,J)** The ToA and HtA of patients with PSD and patients with AD showed in the naming animals-task gait test. **p ≤ 0.01. PSD, post-stroke dementia, AD, Alzheimer’s disease, CoV, coefficient of variance.

In the naming animals-task gait test, participants in the AD group demonstrated significantly smaller ToA and HtA than individuals with PSD (*p* ≤ 0.01, Supplementary Table 2 and Figure 4), except for the single or counting-task gait tests. These two parameters of the postural control domain were robust, showing significant differences after controlling for age, gender, education level, height, and numbers of comorbidities, with or without MMSE scores (Adjusted modal 1 and Adjusted modal 2 of Supplementary Table 2) (*p* ≤ 0.01, Supplementary Table 2). However, no other significant differences in gait parameters were found in the single or dual-task gait tests between individuals with PSD and AD.

**FIGURE 5:**
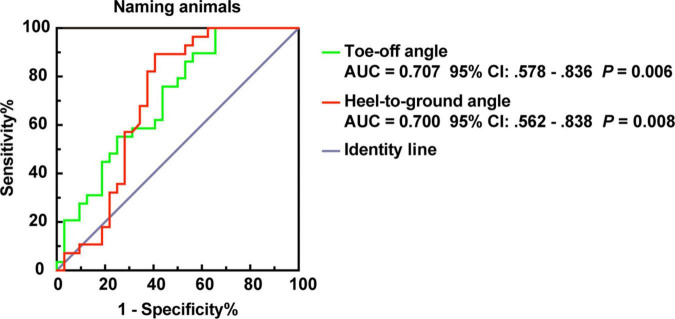
ROC plot for ToA and HtA distinguish PSD and AD of dementia subtypes in the naming animals-task gait test. AUC, area under the curve; CI, confidence interval.

Furthermore, we modeled the above parameters using stepwise logistic regression (Supplementary Table 3). The AUCs showed that ToA and HtA had moderate accuracy for distinguishing AD from patients with PSD (AUC > 0.700, *p* ≤ 0.01, Figure 5).

## Discussion

In the present study, we focused on exploring unique gait markers with high accuracy to distinguish individuals with PSD from those without dementia who had suffered an ischemic stroke 3 months earlier and compared the gait characteristics of patients with PSD with those of patients with AD to understand the unique signatures of gait that reflect the different pathogeneses and pathologies. First, both individuals with PSD and AD showed an impaired pace domain as compared to HCs, while the PSND group did not differ from HCs. Moreover, patients with PSD walked with an impaired rhythm, while patients with AD demonstrated worse postural control during walking than HCs. One main finding of our study is that individuals with PSD had significantly shorter stride length, slower walking speed, and spent a longer percentage of time in the stance phase than individuals with PSND during the single and dual-task gait tests. Increased CoV_SL_ and CoV_ST_ with longer stride time and worse cadence were found only in individuals with PSD during the naming animals-task gait paradigm. On the other hand, significant differences in ToA and HtA during the naming animals-task gait test might allow distinction of individuals with PSD and AD.

Human gait is typically divided into five domains (Lord et al., 2013), four of which were included in our study. Mobility decline with slowing gait is a continuum that co-exists with or even precedes the decline in cognition, which is pervasive and under-recognized in the majority of cognition-motor studies. A mildly reduced pace in individuals with MCI was detected when using dual-task gait paradigms, even though this finding was controversial in a single task-gait paradigm (Cullen et al., 2019; Latorre Román et al., 2020), indicating that mild cognitive decline might influence gait constitution, particularly in condition of more severe cognitive complaints. In addition, several studies have shown that gait slowing occurred in the early stage of cognitive decline and might be a predictor of the risk of progressing to dementia (Montero-Odasso et al., 2017), demonstrating that gait abnormality occurred before a diagnosis of moderate cognitive impairment. Reduced stride length and walking speed have been reported in subjects with AD, particularly those with moderate to severe AD as compared to aged-matched HCs (Mc Ardle et al., 2017). Gait rhythm was generally impaired, as outlined by a few cross-sectional studies, while findings of increasing variability were inconsistent (Boripuntakul et al., 2014; Mc Ardle et al., 2017; Valkanova and Ebmeier, 2017; Pieruccini-Faria et al., 2021). Our results indicated a longer stride time, with higher CoV_ST_ and CoV_SL_, and disturbed pace in individuals with AD performing single or dual-task gait tests, as compared to subjects in the HCs group, which supported the previous findings. The ToA and HtA were smaller in patients with AD than in HCs. PSD, which involves a definite stroke event and subsequent cognitive impairment, is a subtype of VaD but is not equal to VaD. Previously, treatments have focused on the recovery of motor, sensory, visual, or articulatory functions. However, about half of patients with stroke suffer from amnesia and decreased executive capacity, which has not received much attention previously. Cognitive degeneration always indicates a poor prognosis, and it predicts the risk of relapse of stroke (van der Flier et al., 2018). However, if this goes undetected, the best period for therapy may be missed. The cognitive symptoms of PSD, based on one or more infarction lesions in specific brain regions, could sometimes be reversible if diagnosed timely and well-targeted treatment is started early. Thus, the timely distinction of PSD is of great significance. Due to the limited power and accuracy of PSD diagnosis at present, the gait signature, which combines evaluation of cognitive decline and cognition-motor interaction, may facilitate the distinction of PSD from PSND.

Recently, studies have reported that cognitive decrease influences the movement of the post-stroke population. Two cross-sectional studies have shown that disturbed gait and balance were associated with impaired cognition, particularly executive function deficits (Einstad et al., 2021) in post-stroke patients, while a lesion in the right hemisphere might lead to gait complaints (Ursin et al., 2019). Another prospective study found that gait performance was related to executive function when recall over 1 year after mild or moderate acute supratentorial ischemic stroke (Sagnier et al., 2017). A study by Assayag et al. suggested that gait and balance were predictors of cognitive status within 2 years post-stroke (Ben Assayag et al., 2015). However, little had been reported on the gait characteristics of PSD as compared to PSND or individuals with non-stroke cognitive damage, even though the gait characteristics of patients with stroke with severe motor system damage have been reported (Dai et al., 2021). To the best of our knowledge, the gait-based discrimination between individuals with PSD and PSND has not been reported previously. Subjects with stroke enrolled in our study included those with left, right, or bilateral cerebral infarctions, and there were no differences in the lesion side distribution, muscle strength, NIHSS, or mRS scores between the PSD and PSND groups. The subjects in the PSD group demonstrated worse gait, with impaired cognition than those in the PSND group when controlling for baseline demographics. However, after adjusting for MMSE scores, the differences were not significant, suggesting that global cognition plays a crucial role in the gait performance of patients with PSD. Additionally, some gait parameters could identify patients with PSD, as evidenced by the moderate AUCs. Previous studies have revealed that patients with VaD walked with a slower velocity and shorter stride length than patients with AD (Tanaka et al., 1995; Sverdrup et al., 2021). However, we did not find a difference in the pace domain between the PSD and AD groups, even though PSD is a subtype of VaD. Additionally, we found that the smaller ToA or HtA might sensitively identify individuals with AD from individuals with PSD during the naming animals-task gait test. This has not been reported to date, to our knowledge. Whether these two parameters can define other phenotypes of dementia requires further study.

The dual-task gait test is the most popular method to investigate gait in individuals challenged by cognitive complaints and it sensitively detects gait perturbation, because it increases the gap between dementia patients and healthy populations (Al-Yahya et al., 2011; Amboni et al., 2013; Matsuura et al., 2019). Walking while performing a cognitive task will divert more attention, executive function, memory, or even visual and aural resources to complete cognitive tasks as a priority. Thus, during dual-task gait tests, people consciously walk using a more cautious gait, to prevent accidents. Under the naming animals-task condition, participants must distribute their visual capacity and attentional and executive functions simultaneously to the extra task along with walking while the counting-backward task is performed without accessory visual-spatial skills but requires memory. In line with previous evidence, overall gait performance in our study is worse as evidenced by increased CoV_SL_, CoV_ST_, and stride time but decreased cadence of patients with PSD than those of individuals with PSND only in the dual-task gait tests. In addition, the naming animals-task gait test might be a feasible paradigm for exploring the different gait characteristics of individuals with AD and PSD as patients with AD usually have worse visual-spatial capacity. Even so, more rational dual-task gait tests should be attempted, and uniform standard dual-task paradigms should be defined to enhance detection of occult cognitive impairment by testing cognition–motor interaction.

The mechanisms underlying discrete movement weakening in our study could not be simply explained by aging or motor system damage. A well-balanced dynamic gait is a complex achievement achieved not only by the motor system, such as muscle strength, but also by the cognition of individuals, controlled by widespread brain regions that process sensory, attention, executive, visual, and even memory information (Mc Ardle et al., 2017; Allali et al., 2019). An increasing number of studies have suggested that cognition shares some neural structures and pathology with those by which gait is controlled (Mc Ardle et al., 2017). Thus, higher-order region deterioration leading to cognitive decrease might also result in subtle changes in discrete gait characteristics.

To provide insight into the structural imaging-gait correlations, the relationships between functional structure changes and gait performance have been well-studied. Gait velocity provides an overall view of brain function and connection. Impaired pace, including low speed and short stride length, has been associated with decreased gray matter volume in the cortex (Callisaya et al., 2013), basal ganglia, and caudate nucleus (Dumurgier et al., 2012). White matter hyperintensity is also strongly associated with poor attention and executive processing, and these negative changes in the brain also negatively affect gait pace and variability (Wilson et al., 2019), but no association was found between the reduction in white matter volume and pace disturbance (Ezzati et al., 2015).

Different types of dementia show damage in overlapping brain regions and thus these individuals show roughly similar gait pace impairment as compared to age-matched healthy older individuals. The lower velocity and shorter stride length of subjects with AD and PSD in our study support this view. Individuals with VaD mostly show deficits in basal forebrain cholinergic signaling, i.e., a damaged higher-order region conventionally associated with vasculopathy and amyloid deposition in patients with AD (Kalaria, 2002). On the other hand, different subtypes of dementia may have their own typical pathology in specific brain regions, which can result in unique gait characteristics. Frontal and entorhinal cortex atrophy are typical forms of AD. These areas are deemed to process attention, executive function, and control pace (Wilson et al., 2019). On the other hand, posterior cortical atrophy correlates with the initiation of visual-spatial dysfunction (Spasov et al., 2019). This region mainly processes pace, postural control, and cadence in the rhythm domain (Wilson et al., 2019). Hippocampal atrophy, which induces memory decline, may influence rhythm, variability, and postural control (Zimmerman et al., 2009; Beauchet et al., 2019). However, the infarction lesions of patients with stroke in our study were primarily located from the basal ganglia to the periventricular regions, thalamus, pons, and frontal and parietal lobes. The prefrontal cortex-basal ganglia circuit is responsible for gait velocity and step width, along with executive function, while the limbic regions and thalamus may process stride length, width, and cadence, as indicated in previous studies (Callisaya et al., 2013; Takakusaki, 2013; Wilson et al., 2019).

Compared to HCs, we found that the AD and PSD groups showed some unique parameters, providing evidence that differences in deficits in the respective brain regions could induce diverging cognitive symptoms and disease-related gait performance. Based on the comparison of the AD with the PSD group, no difference in the familiar gait parameters was observed, even with dual-task gait tests, except for the ToA and HtA under the naming animals-task condition. Studies on ToA or HtA measured at the moment of initiation or end of the swing phase of dementia patients are scarce, even though largely reported in the patients with PD. We consider that these two parameters belong to the postural control domain because they reflect ankle-related muscle strength, lower limb joint excursion, and foot clearance, which are related to postural instability in patients with PD (Killeen et al., 2017). The ToA and HtA were smaller in the AD group than in the PSD group, indicating that walking while conducting a naming animals-task placed a greater attention demand for joint flexion and limb muscle strength on patients with AD. Muscle strength, flexion, and extension involve walking automaticity produced by the spinal cord, brain stem, cerebellum, and the afferent pathway from the cerebral motor cortex to the brain stem and spinal cord (Clark, 2015). During the naming animals-task, higher central region processing of visual afferent information competes for attentional resources on locomotion, resulting in degraded feedback of automated motion (Clark, 2015). The visual capacity deficit, induced by occipital lobe atrophy, and local neural degeneration exerts a cognitive load on patients with AD during the naming animals-task gait test, while severe impairment of the prefrontal cortex leads to worse executive function. Participants might consciously adjust their stride angle to control postural stability once they become aware that the locomotor system was being challenged. After controlling for demographic covariates and the MMSE score, the statistically significant differences in the ToA and HtA between patients with PSD and patients with AD persisted. The differentiated circuit modulating locomotor and automated motion, or specific cognitive domains, other than global cognition, or peripheral function should be further analyzed to explain this phenomenon. These results suggest that ToA and HtA might be key motion parameters for distinguishing AD from PSD using a standard animals naming-task gait test, with modest accuracy (an intermediate AUC). The stride angle has not been routinely examined in dementia subtypes, and research focusing on the relationship between brain structure and stride angle is sparse. Thus, further studies are needed to investigate these issues.

Amyloid deposition and tau hyperphosphorylation are classical theories for the etiology and pathogenesis of AD (Raz et al., 2016). Abnormal production of amyloid and tau causes toxicity to neurons, negatively changes neuronal activity and synaptic plasticity, activates glial cells and neuroinflammation, induces neuronal death, damages neural circuits, and causes cerebrovascular dysfunction (Raz et al., 2016; Charidimou et al., 2017). In individuals with PSD, a specific brain region suffered ischemia, subsequent neuron death, and inflammation around the lesion. Some studies have indicated that deposition of amyloid and tau also occurred in patients with PSD, and the two subtypes of dementia in older individuals always involve some cerebrovascular malfunction, even though VaD *per se* includes a wider range of vesicular pathological changes (Kalaria, 2002; Emrani et al., 2020). Hence, there is clearly a neuropathological overlap of AD and PSD, which could result in difficulty in identifying subtle differences in some common gait parameters. Additionally, pathological changes in the central nervous system affect the cholinergic system, which plays a critical role in motion and cognition. The basal ganglia afferents to the cerebral cortex are mostly cholinergic neurons, which also modulate hippocampal activity and the frontoparietal networks (Tisch et al., 2004). It has been suggested that AD and PSD both involve acetylcholine signaling disruption and that the cholinergic deficits in AD are related to motor disturbances (Emrani et al., 2020). Previous research has shed light on the fact that acetylcholine esterase inhibitors could decrease the variability and fall incidence of people with mild AD (Montero-Odasso et al., 2009). Whether the cholinergic circuit disturbance might affect the ToA or HtA has not been reported. Further studies should focus on the role of the cholinergic system in stride angles.

On the basis of the lack of differences in gait parameters between individuals with PSND and HCs, we compared the gait characteristics of individuals with PSD and individuals with PSND. We found that individuals with PSD showed deficits in the pace domain, demonstrated as markedly shorter stride length and slower velocity, with a disturbed stance/swing phase ratio, despite a lack of difference in the baseline NIHSS, mRS, muscle strength, and infarction side between the PSD and PSND groups. The difference was robust after controlling for major demographic covariates, numbers of comorbidity, and muscle strength, but did not persist after further adjusting for the MMSE score. This indicated that global cognition was the overriding factor accounting for the gait disturbance in individuals with PSD, whose muscle strength did not differ from that of PSND individuals. The counting-task gait test involves people walking while performing serial subtraction of 1, which requires relatively high numeracy, memory, and attention capacity. The stride length and velocity in the counting-task gait better distinguished individuals with PSD from individuals with PSND, as the AUC increased from 0.738 to 0.806 and from 0.738 to 0.761, respectively, for the single-task gait test. Because velocity reflects global brain function, and stride length and velocity are both highly related to attention and executive function, counting backward might be a means for sensitively detecting attention and memory-associated gait disturbances. As the degree of cognitive loading increased, the variability of gait could discriminate individuals with PSD in the naming animals-task, as demonstrated by an AUC of 0.760 for CoV_SL_ and an AUC of 0.800 for CoV_ST_. The cognitive challenge of the naming animals-task is increased by the additional requirement for visual-spatial skill. However, due to the heterogeneity of the ischemic lesion in the stroke patients in our study, the regional association between specific brain structures and gait characteristics is difficult to depict. Further studies are needed to classify the subtypes of stroke by lesion location and to research the respective gait signature and the interaction of the central structure or function with gait parameters.

Some limitations of our study need to be addressed. First, due to the limited recruitment of patients with AD and patients with PSD, we did not further stratify the patients by the severity of cognitive impairment. Thus, in our study, this population comprised those with mild to moderate dementia. Further studies should focus on more details of gait characteristics in individuals with different levels of cognitive impairment. In addition, the pathological heterogeneity of patients with PSD and patients with AD requires a more rigorous stratification of the underlying central pathology to study the relationship between specific histomorphology and gait domains further.

## Conclusion

In this study, we observed gait impairment in patients with PSD and patients with AD, as compared with matched normal aging controls. Some gait parameters, particularly, the stride length in the counting backward-task and the CoV_ST_ in the naming animals-task gait paradigm could allow the distinction of individuals with PSD from 3-month post-stroke patients without dementia. A smaller ToA and HtA might be characteristic gait features distinguishing subjects with AD from subjects with PSD. Overall, our findings suggest that particular gait characteristics could be non-invasive biomarkers facilitating early diagnosis of individuals with PSD, and could support the use of gait for identification of dementia subtypes, to promote appropriate and early intervention.

## Data Availability Statement

The raw data supporting the conclusions of this article will be made available by the authors, without undue reservation.

## Ethics Statement

The studies involving human participants were reviewed and approved by Medical Ethical Committee of Sir Run Run Shaw Hospital, Zhejiang University School of Medicine. The patients/participants provided their written informed consent to participate in this study.

## Author Contributions

LN and HC designed the study. WL, YiS, and XX performed the study and acquired the data. LN, MC, and XH executed the statistical analysis and made the corresponding tables and figures. YuS and ST interpreted the raw data. LN wrote the manuscript. HC and XYH reviewed and modified the manuscript. All authors contributed to the article and agreed to be accountable for the content of the work.

## Conflict of Interest

The authors declare that the research was conducted in the absence of any commercial or financial relationships that could be construed as a potential conflict of interest.

## Publisher’s Note

All claims expressed in this article are solely those of the authors and do not necessarily represent those of their affiliated organizations, or those of the publisher, the editors and the reviewers. Any product that may be evaluated in this article, or claim that may be made by its manufacturer, is not guaranteed or endorsed by the publisher.

## Abbreviations

PSD: post-stroke dementia
PSND: post-stroke non-dementia
AD: Alzheimer’s disease
HCs: healthy control subjects
CoV: coefficient of variation
ToA: toe-off angle
HtA: heel-to-ground angle
MMSE: Mini-Mental State Examination
MoCA: Montreal Cognitive Assessment
HAMD: 17-item Hamilton Depression Rating Scale
HAMA: Hamilton Anxiety Rating Scale
ADL: activity of daily living
ROC: receiver operating characteristic
AUC: area under the ROC curve
NIHSS: National Institution Health of Stroke Scale
mRS: Modified Rankin Scale
VaD: vascular dementia
MCI: mild cognitive impairment
PD: Parkinson’s disease.

## References

[B1] Allali G. Montembeault M. Brambati S. M. Bherer L. Blumen H. M. Launay C. P. (2019). Brain Structure Covariance Associated With Gait Control in Aging. *J. Gerontol. A Biol. Sci. Med. Sci.* 74 705–713. 10.1093/gerona/gly123 29846517

[B2] Allan L. M. Ballard C. G. Burn D. J. Kenny R. A. (2005). Prevalence and severity of gait disorders in Alzheimer’s and non-Alzheimer’s dementias. *J. Am. Geriatr. Soc.* 53 1681–1687. 10.1111/j.1532-5415.2005.53552.x 16181166

[B3] Al-Yahya E. Dawes H. Smith L. Dennis A. Howells K. Cockburn J. (2011). Cognitive motor interference while walking: a systematic review and meta-analysis. *Neurosci. Biobehav. Rev.* 35 715–728. 10.1016/j.neubiorev.2010.08.008 20833198

[B4] Amboni M. Barone P. Hausdorff J. M. (2013). Cognitive contributions to gait and falls: evidence and implications. *Mov. Dis.* 28 1520–1533. 10.1002/mds.25674 24132840 PMC4119872

[B5] Bayot M. Dujardin K. Tard C. Defebvre L. Bonnet C. T. Allart E. (2018). The interaction between cognition and motor control: A theoretical framework for dual-task interference effects on posture, gait initiation, gait and turning. *Neurophysiol. Clin.* 48 361–375. 10.1016/j.neucli.2018.10.003 30487064

[B6] Beauchet O. Annweiler C. Callisaya M. L. De Cock A. M. Helbostad J. L. Kressig R. W. (2016). Poor Gait Performance and Prediction of Dementia: Results From a Meta-Analysis. *J. Am. Med. Dir. Assoc.* 17 482–490. 10.1016/j.jamda.2015.12.092 26852960 PMC5319598

[B7] Beauchet O. Launay C. P. Sekhon H. Montembeault M. Allali G. (2019). Association of hippocampal volume with gait variability in pre-dementia and dementia stages of Alzheimer disease: Results from a cross-sectional study. *Exp. Gerontol.* 115 55–61. 10.1016/j.exger.2018.11.010 30447261

[B8] Ben Assayag E. Shenhar-Tsarfaty S. Korczyn A. D. Kliper E. Hallevi H. Shopin L. (2015). Gait measures as predictors of poststroke cognitive function: evidence from the TABASCO study. *Stroke* 46 1077–1083. 10.1161/strokeaha.114.007346 25677599

[B9] BoripuntakulS.LordS. R.BrodieM. A.SmithS. T.MethapataraP.WongpakaranN. (2014). Spatial variability during gait initiation while dual tasking is increased in individuals with mild cognitive impairment. *J. Nutr. Health Aging* 18 307–312. 10.1007/s12603-013-0390-3 24626760

[B10] Callisaya M. L. Beare R. Phan T. G. Blizzard L. Thrift A. G. Chen J. (2013). Brain structural change and gait decline: a longitudinal population-based study. *J. Am. Geriatr. Soc.* 61 1074–1079. 10.1111/jgs.12331 23796055

[B11] Charidimou A. Boulouis G. Gurol M. E. Ayata C. Bacskai B. J. Frosch M. P. (2017). Emerging concepts in sporadic cerebral amyloid angiopathy. *Brain* 140 1829–1850. 10.1093/brain/awx047 28334869 PMC6059159

[B12] Cheng Q. Wu M. Wu Y. Hu Y. Kwapong W. R. Shi X. (2020). Weaker Braking Force, A New Marker of Worse Gait Stability in Alzheimer Disease. *Front. Aging Neurosci.* 12:554168. 10.3389/fnagi.2020.554168 33024432 PMC7516124

[B13] Clark D. J. (2015). Automaticity of walking: functional significance, mechanisms, measurement and rehabilitation strategies. *Front. Hum. Neurosci.* 9:246. 10.3389/fnhum.2015.00246 25999838 PMC4419715

[B14] Cognitive Impairment Committee Nb, Chinese Medical Doctor Association. (2019). A guideline for the diagnosis and treatment of Chinese vascular cognitive impairment in 2019. *Chin. Med. J.* 99 2737–2744.

[B15] Cohen J. A. Verghese J. Zwerling J. L. (2016). Cognition and gait in older people. *Maturitas* 93 73–77. 10.1016/j.maturitas.2016.05.005 27240713

[B16] Cullen S. Borrie M. Carroll S. Sarquis-Adamson Y. Pieruccini-Faria F. McKay S. (2019). Are Cognitive Subtypes Associated with Dual-Task Gait Performance in a Clinical Setting? *J. Alzheimers Dis.* 71 S57–S64. 10.3233/jad-181196 31322559

[B17] Dai S. Piscicelli C. Clarac E. Baciu M. Hommel M. Pérennou D. (2021). Balance, Lateropulsion, and Gait Disorders in Subacute Stroke. *Neurology* 96 e2147–e2159. 10.1212/wnl.0000000000011152 33177223

[B18] Dumurgier J. Crivello F. Mazoyer B. Ahmed I. Tavernier B. Grabli D. (2012). MRI atrophy of the caudate nucleus and slower walking speed in the elderly. *Neuroimage* 60 871–878. 10.1016/j.neuroimage.2012.01.102 22305950

[B19] Einstad M. S. Saltvedt I. Lydersen S. Ursin M. H. Munthe-Kaas R. Ihle-Hansen H. (2021). Associations between post-stroke motor and cognitive function: a cross-sectional study. *BMC Geriatr.* 21:103. 10.1186/s12877-021-02055-7 33546620 PMC7863272

[B20] Emrani S. Lamar M. Price C. C. Wasserman V. Matusz E. Au R. (2020). Alzheimer’s/Vascular Spectrum Dementia: Classification in Addition to Diagnosis. *J. Alzheimers Dis.* 73 63–71. 10.3233/jad-190654 31815693

[B21] Ezzati A. Katz M. J. Lipton M. L. Lipton R. B. Verghese J. (2015). The association of brain structure with gait velocity in older adults: a quantitative volumetric analysis of brain MRI. *Neuroradiology* 57 851–861. 10.1007/s00234-015-1536-2 25921321 PMC4553137

[B22] Ghoraani B. Boettcher L. N. Hssayeni M. D. Rosenfeld A. Tolea M. I. Galvin J. E. (2021). Detection of Mild Cognitive Impairment and Alzheimer’s Disease using Dual-task Gait Assessments and Machine Learning. *Biomed. Signal Process Control* 64:102249. 10.1016/j.bspc.2020.102249 33123214 PMC7591132

[B23] Gillain S. Warzee E. Lekeu F. Wojtasik V. Maquet D. Croisier J. L. (2009). The value of instrumental gait analysis in elderly healthy, MCI or Alzheimer’s disease subjects and a comparison with other clinical tests used in single and dual-task conditions. *Ann. Phys. Rehabil. Med.* 52 453–474. 10.1016/j.rehab.2008.10.004 19525161

[B24] Ismail Z. Black S. E. Camicioli R. Chertkow H. Herrmann N. Laforce R. Jr. (2020). Recommendations of the 5th Canadian Consensus Conference on the diagnosis and treatment of dementia. *Alzheimers Dement.* 16 1182–1195. 10.1002/alz.12105 32725777 PMC7984031

[B25] Kalaria R. (2002). Similarities between Alzheimer’s disease and vascular dementia. *J. Neurol. Sci.* 203-204 29–34. 10.1016/s0022-510x(02)00256-3 12417353

[B26] Khachaturian Z. S. (2011). Revised criteria for diagnosis of Alzheimer’s disease: National Institute on Aging-Alzheimer’s Association diagnostic guidelines for Alzheimer’s disease. *Alzheimers Dement.* 7 253–256. 10.1016/j.jalz.2011.04.003 21575869

[B27] Killeen T. Easthope C. S. Demkó L. Filli L. Lõrincz L. Linnebank M. (2017). Minimum toe clearance: probing the neural control of locomotion. *Sci. Rep.* 7:1922. 10.1038/s41598-017-02189-y 28507300 PMC5432520

[B28] Latorre Román P. Muñoz Jiménez M. Salas Sánchez J. Consuegra González P. Moreno Del Castillo R. Herrador Sánchez J. A. (2020). Complex Gait Is Related to Cognitive Functioning in Older People: A Cross-Sectional Study Providing an Innovative Test. *Gerontology* 66 401–408. 10.1159/000508245 32623430

[B29] Li H. Jia J. Yang Z. (2016). Mini-Mental State Examination in Elderly Chinese: A Population-Based Normative Study. *J. Alzheimers Dis.* 53 487–496. 10.3233/jad-160119 27163822

[B30] Lord S. Galna B. Verghese J. Coleman S. Burn D. Rochester L. (2013). Independent domains of gait in older adults and associated motor and nonmotor attributes: validation of a factor analysis approach. *J. Gerontol. A Biol. Sci. Med. Sci.* 68 820–827. 10.1093/gerona/gls255 23250001

[B31] Lu J. Li D. Li F. Zhou A. Wang F. Zuo X. (2011). Montreal cognitive assessment in detecting cognitive impairment in Chinese elderly individuals: a population-based study. *J. Geriatr. Psychiatry Neurol.* 24 184–190. 10.1177/0891988711422528 22228824

[B32] Maquet D. Lekeu F. Warzee E. Gillain S. Wojtasik V. Salmon E. (2010). Gait analysis in elderly adult patients with mild cognitive impairment and patients with mild Alzheimer’s disease: simple versus dual task: a preliminary report. *Clin. Physiol. Funct. Imaging* 30 51–56. 10.1111/j.1475-097X.2009.00903.x 19799614

[B33] Matsuura T. Sakashita K. Grushnikov A. Okura F. Mitsugami I. Yagi Y. (2019). Statistical Analysis of Dual-task Gait Characteristics for Cognitive Score Estimation. *Sci. Rep.* 9:19927. 10.1038/s41598-019-56485-w 31882727 PMC6934525

[B34] Mc Ardle R. Galna B. Donaghy P. Thomas A. Rochester L. (2019). Do Alzheimer’s and Lewy body disease have discrete pathological signatures of gait? *Alzheimers Dement.* 15 1367–1377. 10.1016/j.jalz.2019.06.4953 31548122

[B35] Mc Ardle R. Morris R. Wilson J. Galna B. Thomas A. J. Rochester L. (2017). What Can Quantitative Gait Analysis Tell Us about Dementia and Its Subtypes? A Structured Review. *J. Alzheimers Dis.* 60 1295–1312. 10.3233/jad-170541 29036826

[B36] McCarten J. R. Anderson P. Kuskowski M. A. McPherson S. E. Borson S. (2011). Screening for cognitive impairment in an elderly veteran population: acceptability and results using different versions of the Mini-Cog. *J. Am. Geriatr. Soc.* 59 309–313. 10.1111/j.1532-5415.2010.03249.x 21314650

[B37] Montero-Odasso M. M. Sarquis-Adamson Y. Speechley M. Borrie M. J. Hachinski V. C. Wells J. (2017). Association of Dual-Task Gait With Incident Dementia in Mild Cognitive Impairment: Results From the Gait and Brain Study. *JAMA Neurol.* 74 857–865. 10.1001/jamaneurol.2017.0643 28505243 PMC5710533

[B38] Montero-Odasso M. Wells J. Borrie M. (2009). Can cognitive enhancers reduce the risk of falls in people with dementia? An open-label study with controls. *J. Am. Geriatr. Soc.* 57 359–360. 10.1111/j.1532-5415.2009.02085.x 19207156 PMC5017867

[B39] Morris R. Lord S. Bunce J. Burn D. Rochester L. (2016). Gait and cognition: Mapping the global and discrete relationships in ageing and neurodegenerative disease. *Neurosci. Biobehav. Rev.* 64 326–345. 10.1016/j.neubiorev.2016.02.012 26915926

[B40] Noce Kirkwood R. de Souza Moreira B. Mingoti S. A. Faria B. F. Sampaio R. F. Alves Resende R. (2018). The slowing down phenomenon: What is the age of major gait velocity decline? *Maturitas* 115 31–36. 10.1016/j.maturitas.2018.06.005 30049344

[B41] Pieruccini-Faria F. Black S. E. Masellis M. Smith E. E. Almeida Q. J. Li K. Z. H. (2021). Gait variability across neurodegenerative and cognitive disorders: Results from the Canadian Consortium of Neurodegeneration in Aging (CCNA) and the Gait and Brain Study. *Alzheimers Dement.* 2021:12298. 10.1002/alz.12298 33590967 PMC8451764

[B42] Rasmussen L. J. H. Caspi A. Ambler A. Broadbent J. M. Cohen H. J. d’Arbeloff T. (2019). Association of Neurocognitive and Physical Function With Gait Speed in Midlife. *JAMA Netw. Open* 2:e1913123. 10.1001/jamanetworkopen.2019.13123 31603488 PMC6804027

[B43] Raz L. Knoefel J. Bhaskar K. (2016). The neuropathology and cerebrovascular mechanisms of dementia. *J. Cereb. Blood Flow Metab.* 36 172–186. 10.1038/jcbfm.2015.164 26174330 PMC4758551

[B44] Sagnier S. Renou P. Olindo S. Debruxelles S. Poli M. Rouanet F. (2017). Gait Change Is Associated with Cognitive Outcome after an Acute Ischemic Stroke. *Front. Aging Neurosci.* 9:153. 10.3389/fnagi.2017.00153 28572768 PMC5435741

[B45] Sanders L. M. J. Hortobágyi T. Karssemeijer E. G. A. Van der Zee E. A. Scherder E. J. A. van Heuvelen M. J. G. (2020). Effects of low- and high-intensity physical exercise on physical and cognitive function in older persons with dementia: a randomized controlled trial. *Alzheimers Res. Ther.* 12:28. 10.1186/s13195-020-00597-3 32192537 PMC7082953

[B46] Schott J. M. Reiniger L. Thom M. Holton J. L. Grieve J. Brandner S. (2010). Brain biopsy in dementia: clinical indications and diagnostic approach. *Acta Neuropathol.* 120 327–341. 10.1007/s00401-010-0721-y 20640903

[B47] Skrobot O. A. Black S. E. Chen C. DeCarli C. Erkinjuntti T. Ford G. A. (2018). Progress toward standardized diagnosis of vascular cognitive impairment: Guidelines from the Vascular Impairment of Cognition Classification Consensus Study. *Alzheimers Dement.* 14 280–292. 10.1016/j.jalz.2017.09.007 29055812

[B48] Spasov S. Passamonti L. Duggento A. Liò P. Toschi N. (2019). A parameter-efficient deep learning approach to predict conversion from mild cognitive impairment to Alzheimer’s disease. *Neuroimage* 189 276–287. 10.1016/j.neuroimage.2019.01.031 30654174

[B49] Sverdrup K. Selbæk G. Bergh S. Strand B. H. Thingstad P. Skjellegrind H. K. (2021). Physical performance across the cognitive spectrum and between dementia subtypes in a population-based sample of older adults: The HUNT study. *Arch. Gerontol. Geriatr.* 95:104400. 10.1016/j.archger.2021.104400 33798998

[B50] Takakusaki K. (2013). Neurophysiology of gait: from the spinal cord to the frontal lobe. *Mov. Dis.* 28 1483–1491. 10.1002/mds.25669 24132836

[B51] Tanaka A. Okuzumi H. Kobayashi I. Murai N. Meguro K. Nakamura T. (1995). Gait disturbance of patients with vascular and Alzheimer-type dementias. *Percept. Mot. Skills* 80(3 Pt 1), 735–738. 10.2466/pms.1995.80.3.735 7567389

[B52] Tian Q. Chastan N. Bair W. N. Resnick S. M. Ferrucci L. Studenski S. A. (2017). The brain map of gait variability in aging, cognitive impairment and dementia-A systematic review. *Neurosci. Biobehav. Rev.* 74(Pt A), 149–162. 10.1016/j.neubiorev.2017.01.020 28115194 PMC5303129

[B53] Tisch S. Silberstein P. Limousin-Dowsey P. Jahanshahi M. (2004). The basal ganglia: anatomy, physiology, and pharmacology. *Psychiatr. Clin. North Am.* 27 757–799. 10.1016/j.psc.2004.06.004 15550292

[B54] Ursin M. H. Bergland A. Fure B. Thommessen B. Hagberg G. Øksengård A. R. (2019). Gait and balance one year after stroke; relationships with lesion side, subtypes of cognitive impairment and neuroimaging findings-a longitudinal, cohort study. *Physiotherapy* 105 254–261. 10.1016/j.physio.2018.07.007 30340837

[B55] Valkanova V. Ebmeier K. P. (2017). What can gait tell us about dementia? Review of epidemiological and neuropsychological evidence. *Gait Posture* 53 215–223. 10.1016/j.gaitpost.2017.01.024 28222369

[B56] van der Flier W. M. Skoog I. Schneider J. A. Pantoni L. Mok V. Chen C. L. H. (2018). Vascular cognitive impairment. *Nat. Rev. Dis. Primers* 4:18003. 10.1038/nrdp.2018.3 29446769

[B57] WHO (2018). *The Global Dementia Observatory Reference Guide version1.1.* Geneva: WHO.

[B58] Wilson J. Allcock L. Mc Ardle R. Taylor J. P. Rochester L. (2019). The neural correlates of discrete gait characteristics in ageing: A structured review. *Neurosci. Biobehav. Rev.* 100 344–369. 10.1016/j.neubiorev.2018.12.017 30552912 PMC6565843

[B59] Zimmerman M. E. Lipton R. B. Pan J. W. Hetherington H. P. Verghese J. (2009). MRI- and MRS-derived hippocampal correlates of quantitative locomotor function in older adults. *Brain Res.* 1291 73–81. 10.1016/j.brainres.2009.07.043 19631621 PMC2747520

