# Supplementary material for: Pathological Gait Signatures of Post-stroke Dementia With Toe-Off and Heel-to-Ground Angles Discriminate From Alzheimer’s Disease
Source: Front Aging Neurosci. 2021 Nov 18;13:766884. doi: 10.3389/fnagi.2021.766884 (PMC8638706; doi:10.3389/fnagi.2021.766884)
Supplement: Supplementary file 1 [file Data_Sheet_1.docx]

Supplementary Material

# Supplementary Tables and Figures

## Supplementary Tables

| **Supplemental Table S1.**  **Stroke characteristics of patients with post-stroke** | | | | |  |
| --- | --- | --- | --- | --- | --- |
|  |  |  |  |  |  |
|  | **PSND** | **PSD** | **U /** **ꭓ2** | ***P*** |  |
| **NIHSS** | n = 32 | n = 32 |  |  |  |
| Baseline, median (min–max) | 1 (0–5) | 1 (0–5) | 617.50 | 0.141 |  |
| 3 months, median (min–max) | 0 (0–2) | 0 (0–2) | 561.00 | 0.347 |  |
| **mRS** |  |  |  |  |  |
| Baseline, median (min–max) | 0 (0–2) | 0 (0–2) | 569.50 | 0.283 |  |
| 3 months, median (min–max) | 0 (0–1) | 0 (0–1) | 528.00 | 0.644 |  |
| **Muscle strength (grade), No. (%)** | |  | 0.739 | 0.691 |  |
| 5 of two sides | 20 (62.5) | 23 (71.9) |  |  |  |
| 5- of one or two sides | 10 (31.3) | 7 (21.9) |  |  |  |
| 4+ of one side | 2 (6.3) | 2 (6.3) |  |  |  |
| **Lesion side, No. (%)** |  |  | 1.173 | 0.759 |  |
| Left | 16 (50.0) | 14 (43.8) |  |  |  |
| Right | 13 (40.6) | 12 (37.5) |  |  |  |
| Bilateral | 2 (6.3) | 4 (12.5) |  |  |  |
| Others | 1 (3.1) | 2 (6.3) |  |  |  |

Note: Data displayed as median (min – max) were used Mann-Whitney U test, while ranked data were used the chi-square test. Abbreviation: PSND, post-stroke non-dementia, PSD, post-stroke dementia, NIHSS, National Institution Health of Stroke Scale, mRS, Modified Rankin Scale.

| **Supplemental Table S2.**  **Comparison of gait characteristics of patients with PSD and patients with AD** | | | | | | |
| --- | --- | --- | --- | --- | --- | --- |
|  |  |  |  |  |  |  |
|  | **Unadjusted model** | | **Adjusted model 1** | | **Adjusted model 2** | |
|  | **t / U** | ***P*** | **F / ꭓ^2^** | ***P*** | **F / ꭓ^2^** | ***P*** |
| **Pace** |  |  |  |  |  |  |
| **Stride length (m)** |  |  |  |  |  |  |
| Single-task | 0.661 | 0.511 | 1.401 | 0.242 | 4.022 | 0.050 |
| Counting | 0.264 | 0.792 | 1.218 | 0.274 | 2.867 | 0.050 |
| Naming animals | -1.276 | 0.207 | 0.414 | 0.523 | 0.161 | 0.690 |
| **Velocity (m/s)** |  |  |  |  |  |  |
| Single-task | 0.447 | 0.657 | 0.160 | 0.691 | 1.499 | 0.226 |
| Counting | 0.095 | 0.925 | 0.104 | 0.749 | 1.419 | 0.239 |
| Naming animals | -0.992 | 0.326 | 0.177 | 0.676 | 0.259 | 0.613 |
| **Variability** |  |  |  |  |  |  |
| **CoV_SL_** |  |  |  |  |  |  |
| Single-task | 356.000 | 0.165 | 4.408 | 0.036 | 2.136 | 0.144 |
| Counting | 0.761 | 0.450 | 0.971 | 0.329 | 0.283 | 0.597 |
| Naming animals | 1.991 | 0.051 | 4.780 | **0.033** | 1.139 | 0.291 |
| **CoV_ST_** |  |  |  |  |  |  |
| Single-task | 377.000 | 0.503 | 0.120 | 0.729 | 0.001 | 0.974 |
| Counting | -0.969 | 0.337 | 1.060 | 0.308 | 2.999 | 0.090 |
| Naming animals | 347.000 | 0.354 | 2.377 | 0.123 | 2.118 | 0.146 |
| **Rhythm** |  |  |  |  |  |  |
| **Stride time (s)** |  |  |  |  |  |  |
| Single-task | 1.126 | 0.265 | 4.977 | **0.030** | 3.017 | 0.088 |
| Counting | 476.000 | 0.949 | 1.389 | 0.239 | 0.322 | 0.571 |
| Naming animals | -0.281 | 0.780 | 0.309 | 0.581 | 0.763 | 0.387 |
| **Cadence (steps/min)** |  |  |  |  |  |  |
| Single-task | -0.375 | 0.709 | 1.840 | 0.180 | 0.950 | 0.334 |
| Counting | 477.500 | 0.799 | 0.413 | 0.520 | 0.017 | 0.897 |
| Naming animals | -0.176 | 0.861 | 0.011 | 0.916 | 1.011 | 0.319 |
| **Stance phase (%)** |  |  |  |  |  |  |
| Single-task | -1.328 | 0.189 | 1.488 | 0.228 | 1.984 | 0.165 |
| Counting | -1.524 | 0.133 | 3.438 | 0.069 | 5.742 | **0.020** |
| Naming animals | -0.773 | 0.443 | 1.566 | 0.216 | 7.718 | **0.008** |
| **Swing phase (%)** |  |  |  |  |  |  |
| Single-task | 1.341 | 0.185 | 1.523 | 0.222 | 2.026 | 0.160 |
| Counting | 1.503 | 0.138 | 3.380 | 0.071 | 5.678 | **0.021** |
| Naming animal | 0.770 | 0.445 | 1.574 | 0.215 | 7.761 | **0.007** |
| **Postural control** |  |  |  |  |  |  |
| **Toe-off angle (∘)** | |  |  |  |  |  |
| Single-task | 551.500 | 0.445 | 4.018 | 0.045 | 2.764 | 0.096 |
| Counting | 561.500 | 0.368 | 4.150 | 0.042 | 2.661 | 0.103 |
| Naming animals | 656.000 | **0.006** | 16.520 | **< 0.001** | 12.163 | **< 0.001** |
| **Heel-to-ground angle (∘)** | |  |  |  |  |  |
| Single-task | 460.000 | 0.621 | 0.134 | 0.715 | 0.129 | 0.720 |
| Counting | 466.500 | 0.685 | 0.465 | 0.495 | 0.384 | 0.536 |
| Naming animals | -3.441 | **0.001** | 9.202 | **0.004** | 5.066 | **0.029** |

Note: Normal distributed data were used student *t*-test, the adjusted modals of which were used general linear models, otherwise used Mann-Whitney U test, and control primary covariates by generalized linear models. The significant difference confined by *P* < 0.05. Bold values highlight the significant differences between two groups. Abbreviation: PSD, post-stroke dementia, AD, Alzheimer’s disease.

Adjusted model 1: controlling for age, gender, education levels, height, and numbers of comorbidity.

Adjusted model 2: controlling for age, gender, education levels, height, numbers of comorbidity, and MMSE.

| **Supplemental Table S3.**  **Logistic regression of primary gait parameters to identify patients with PSD from patients with AD in the naming animals-task gait test** | | | | | | | | | |  |
| --- | --- | --- | --- | --- | --- | --- | --- | --- | --- | --- |
|  |  |  |  |  |  |  |  |  |  |  |
|  | **Unadjusted modal** | | | **Adjusted modal 1** | | | **Adjusted modal 2** | | |  |
|  | **OR** | **95% CI** | ***P*** | **OR** | **95% CI** | ***P*** | **OR** | **95% CI** | ***P*** |  |
| CoV_SL_ | 0.842 | 0.72-0.99 | **0.034** | 0.806 | 0.66-0.99 | **0.037** | 0.896 | 0.73-1.10 | 0.288 |  |
| CoV_ST_ | 0.744 | 0.58-0.95 | **0.017** | 0.660 | 0.48-0.92 | **0.013** | 0.730 | 0.51-1.04 | 0.084 |  |
| Stance phase | 1.058 | 0.92-1.22 | 0.437 | 1.121 | 0.92-1.37 | 0.260 | 1.501 | 1.11-2.04 | **0.009** |  |
| Swing phase | 0.945 | 0.82-1.09 | 0.438 | 0.892 | 0.73-1.09 | 0.258 | 0.666 | 0.49-0.90 | **0.009** |  |
| ToA | 1.046 | 1.01-1.09 | **0.025** | 1.089 | 1.02-1.16 | **0.009** | 1.093 | 1.02-1.18 | **0.019** |  |
| HtA | 1.092 | 1.02-1.16 | **0.007** | 1.111 | 1.02-1.21 | **0.017** | 1.107 | 1.004-1.22 | **0.040** |  |

Note: Bold values highlight the significant differences between two groups. Abbreviation: PSD, post-stroke dementia, AD, Alzheimer’s disease.

Adjusted model 1: controlling for age, gender, education levels, height, muscle strength, and numbers of comorbidity.

Adjusted model 2: controlling for age, gender, education levels, height, muscle strength, numbers of comorbidity, and MMSE.

## Supplementary Figures


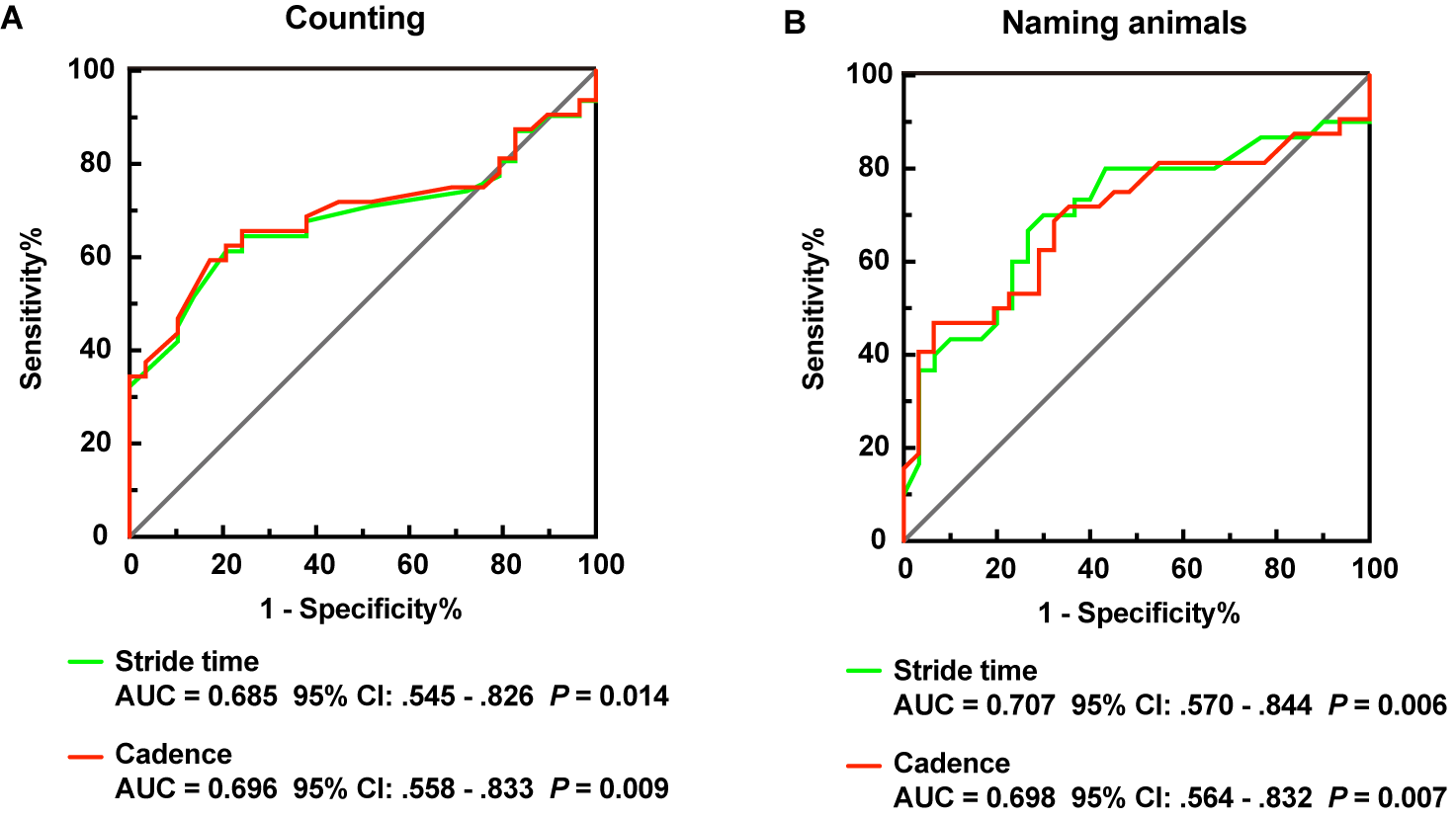


**Supplementary Figure 1.** ROC plot and AUC of the rhythm domain that identify PSD from PSND individuals. **(A)**. The counting-task gait test. (**B)**. The naming animal-task gait test. Abbreviations: AUC, area under the curve, CI, confidence interval, PSD, post-stroke dementia, PSND, post-stroke non-dementia.
